# A Case Report of Irregular Narrow Complex Tachycardia: Atrioventricular Nodal Re-entrant Tachycardia with a Lower Common Pathway Wenckebach

**DOI:** 10.19102/icrm.2026.17083

**Published:** 2026-08-15

**Authors:** Sudipta Mondal, Rajesh Nanda, Nadeem Afroz Muslim

**Affiliations:** 1 Department of Cardiology, The Mission Hospital, Durgapur, India; 2 Department of Cardiology, Neotia Getwel Multispecialty Hospital, Siliguri, India

**Keywords:** Atrioventricular nodal re-entrant tachycardia, AVNRT, infra-Hisian, supra-Hisian, Wenckebach

## Abstract

Previous reports have established the occurrence of atrioventricular (AV) nodal re-entrant tachycardia (AVNRT) exhibiting fixed AV ratios (most commonly 1:1, but also 2:1 or higher grade) or fixed/alternating ventriculoatrial ratios (eg, 1:1, 2:1, 3:2, 4:3, or higher grade). In contrast, the manifestation of an irregularly irregular rhythm during sustained AVNRT combined with a lower common pathway Wenckebach periodicity is exceedingly rare. We formally present a case demonstrating this unusual and complex electrophysiological phenomenon.

## Introduction

Atrioventricular (AV) nodal re-entrant tachycardia (AVNRT) with fixed AV ratios (eg, 1:1 most commonly, 2:1, or higher grade) or fixed/alternating ventriculoatrial (VA) ratios (eg, 1:1 most commonly, 2:1, 3:2, 4:3, or higher grade) has been reported earlier.^1–3^ However, an irregularly irregular rhythm in an ongoing AVNRT with a lower common pathway (LCP) Wenckebach has rarely been reported. We present a similar rare case of irregularly irregular AVNRT.

## Case presentation

**Figure 1:**
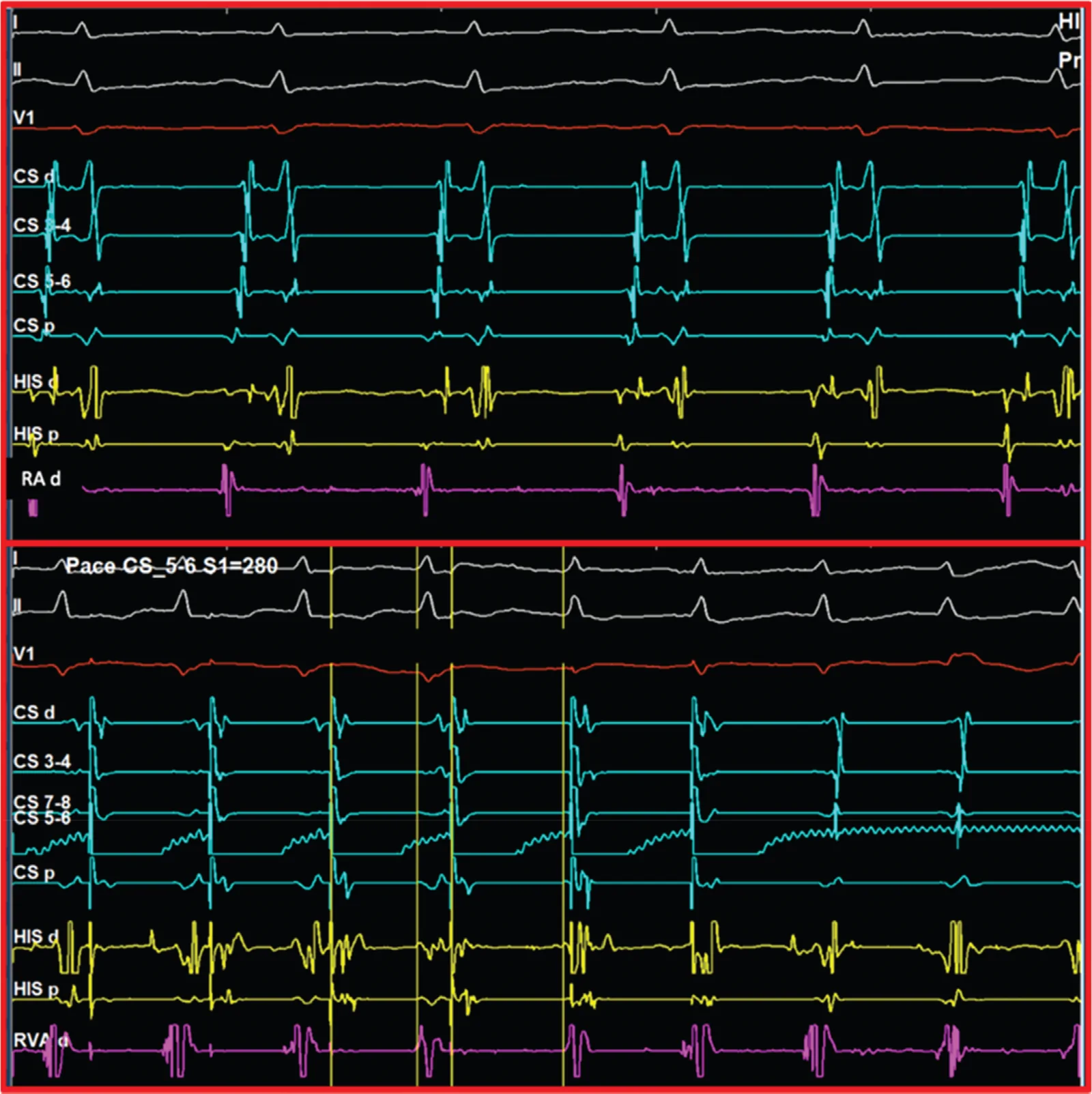
Electrocardiogram and intracavitary electrograms: Baseline and induction of tachycardia.

**Figure 2:**
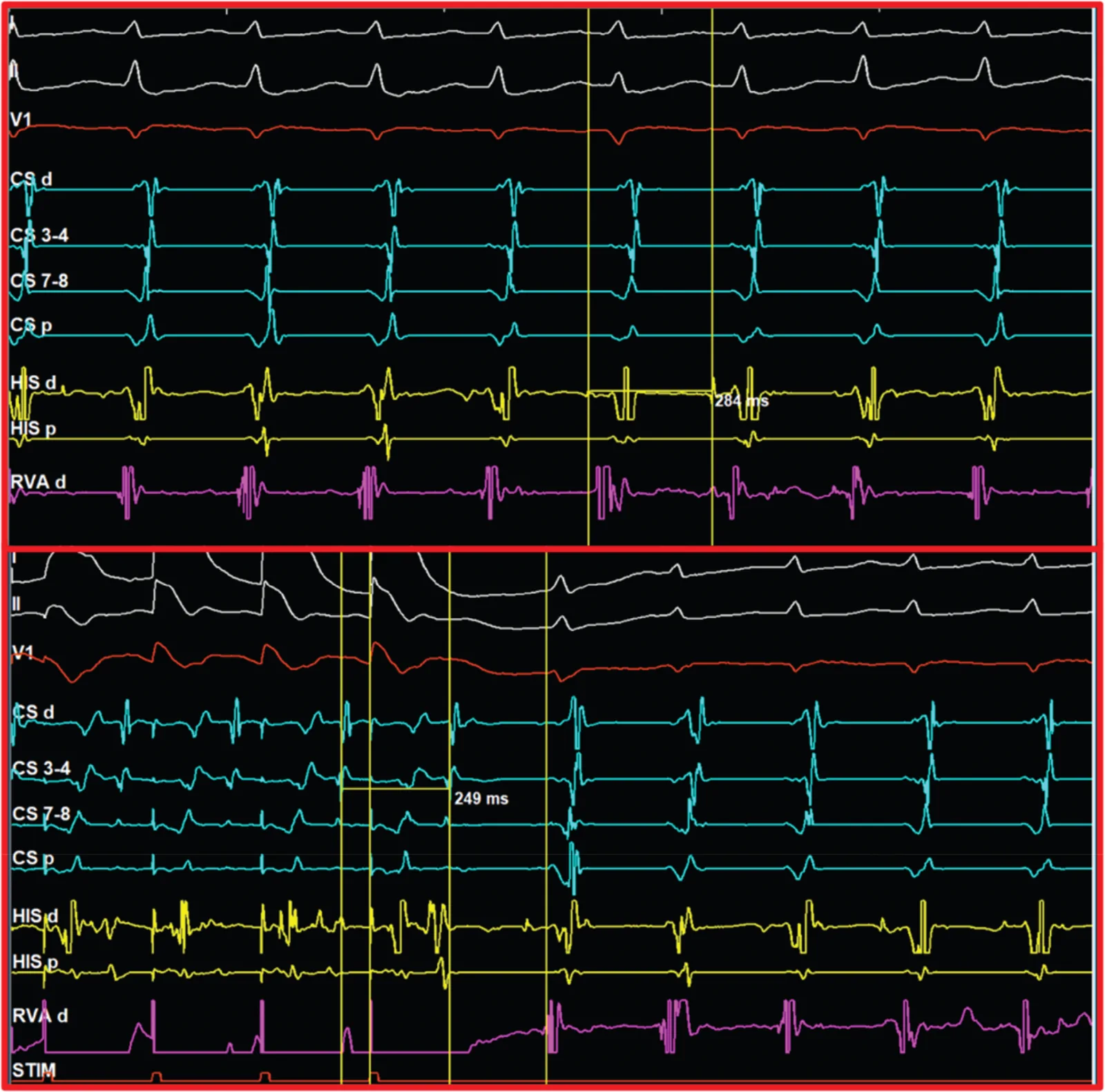
Electrocardiogram and intracavitary electrograms: Tachycardia and ventricular entrainment suggestive of typical atrioventricular nodal re-entrant tachycardia.

**Figure 3:**
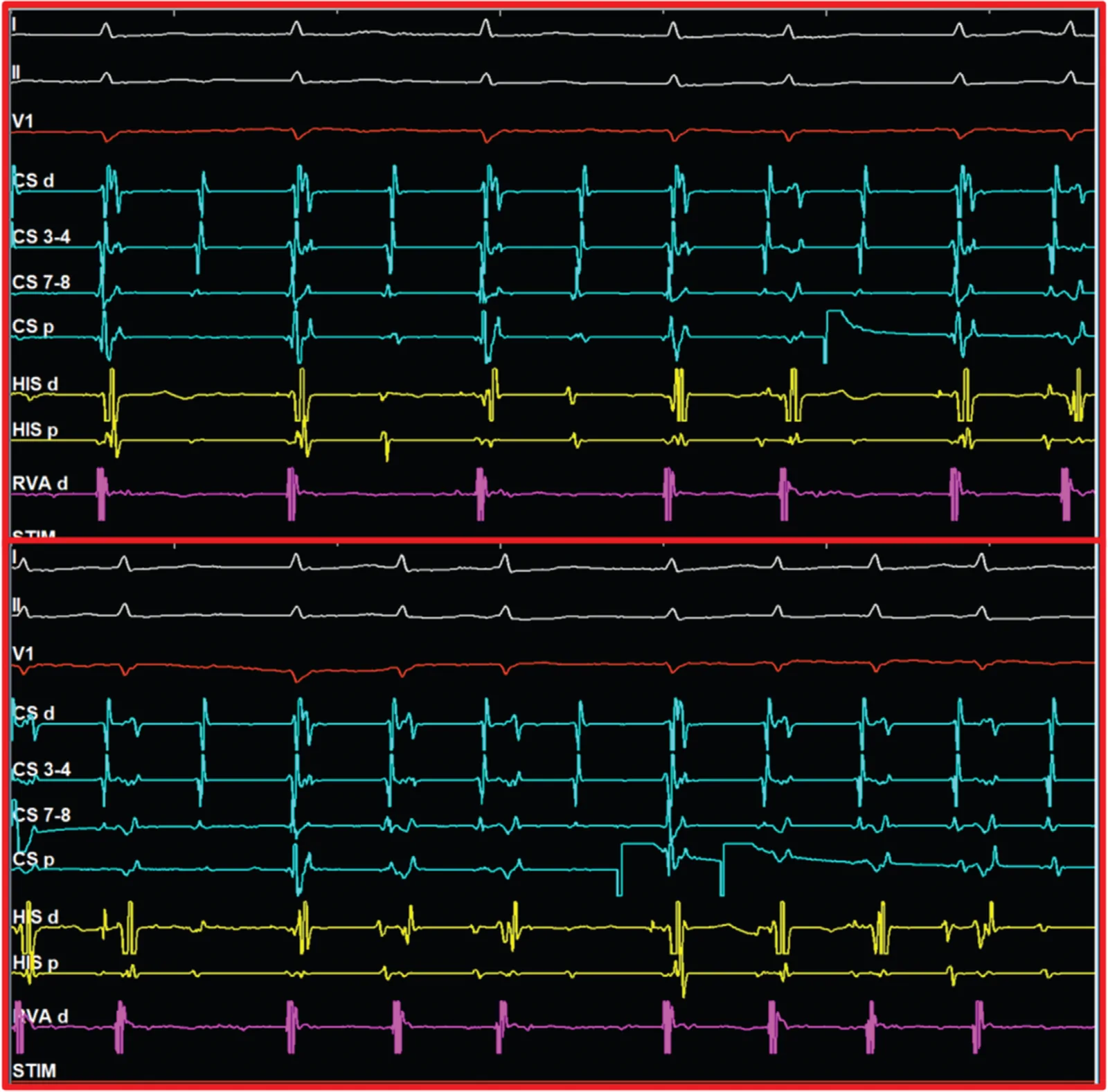
Electrocardiogram and intracavitary electrograms: Narrow complex tachycardia in the index patient (spontaneous transition from a regular narrow QRS tachycardia [NCT] to an irregular NCT exhibiting Wenckebach periodicity).

A 46-year-old man presented with recurrent paroxysmal narrow complex tachycardia responsive to intravenous adenosine. He had a structurally normal heart without any specific cardiac comorbidities. An electrophysiological study revealed atrio–His (A–H) and His–ventricular (H–V) intervals of 62 and 42 ms **(Figure 1A)**, respectively (sinus cycle length, 750 ms). During incremental atrial pacing, a narrow complex tachycardia was induced, with a tachycardia cycle length of 284 ms **(Figures 1B and 2A)**. However, a variable R–R interval with intermittent dropped beats was noted spontaneously **(Figure 3)**.

**Figure 4:**
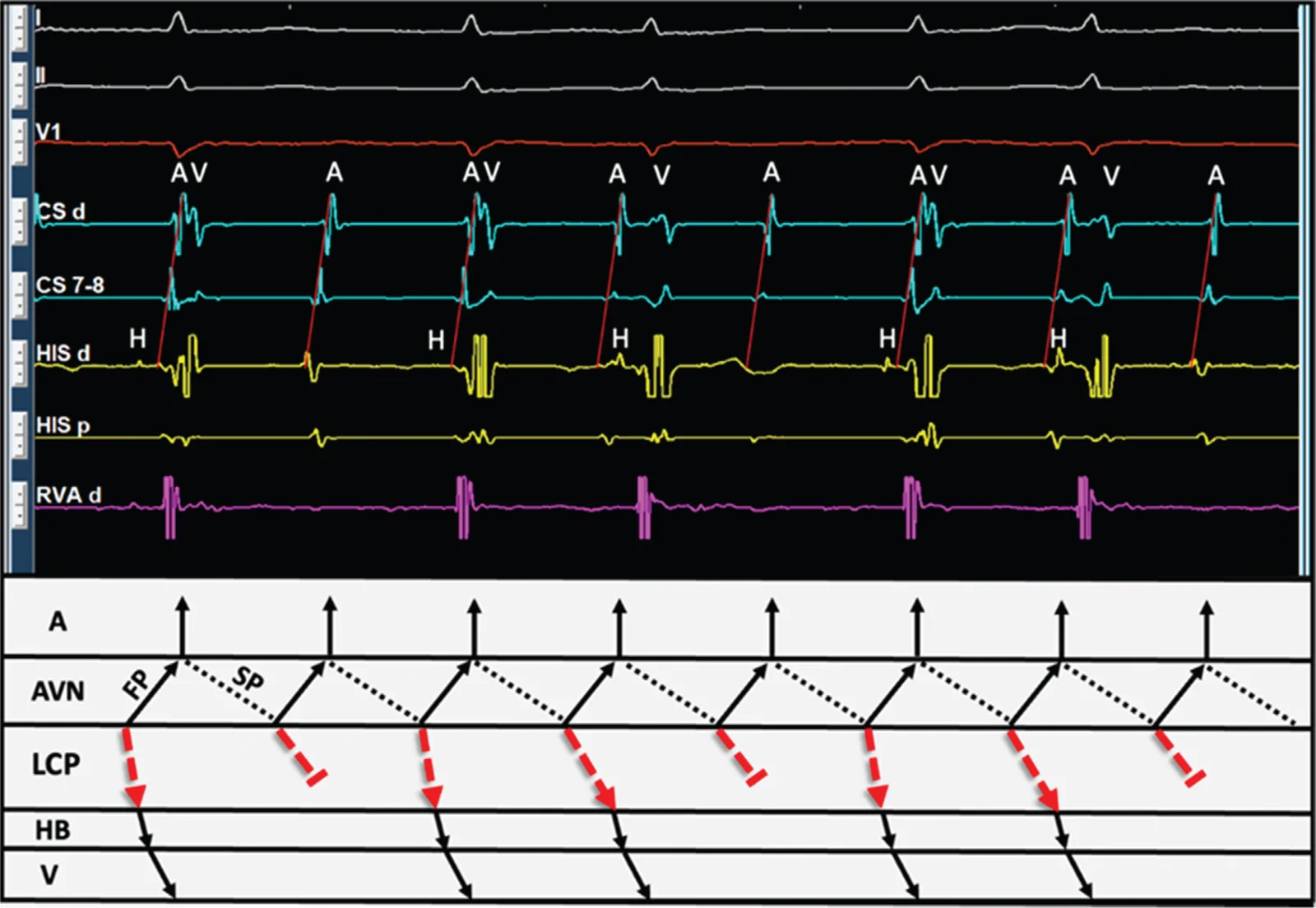
Electrocardiogram and intracavitary electrograms: In the cardiac cycle immediately preceding the dropped beat, the His-bundle (HB) potential (HBP) was consistently observed after the atrial deflection. This configuration signifies a maximal atrio–His (AH) interval prolongation, which was sufficient to place the HBP subsequent to the retrograde atrial activation. This sequence suggests that the maximum possible A–H delay had been achieved before the conduction failure. The observed progressive A–H interval prolongation, which defines the Wenckebach periodicity, must be attributed to a conduction decrement within the lower common pathway (LCP) rather than the antegrade slow pathway (SP). Had the Wenckebach phenomenon occurred in the antegrade SP, the resultant block would have interrupted the forward limb of the re-entrant circuit, thereby terminating the tachycardia. The sustained nature of the rhythm, despite the progressively delaying conduction, confirms that the site of block lies distally in the LCP (infranodal, suprahisian Wenckebach), allowing the re-entrant circuit to persist despite having an LCP block. Immediately following this LCP block, the HBP timing reverted, appearing before the atrial signal as seen in conventional atrioventricular nodal re-entrant tachycardia (AVNRT). The progressive delay observed in the LCP conduction resulted in the HBP exhibiting temporal overlap with the atrial activity within the His catheter recording. This overlap significantly impeded the clear discrimination and measurement of the HBP from the atrial signal in the His catheter. The slanted red lines indicate the atrial activation sequence, which should not change during an ongoing AVNRT. Note the onset of atrial deflections in the HB electrograms. *Abbreviations:* A, atrium; AVN, atrioventricular node; FP, fast pathway; V, ventricle.

This tachyarrhythmia presented with a unique pattern, characterized by the spontaneous transition from a regular narrow QRS tachycardia (NCT) to an irregular NCT exhibiting Wenckebach periodicity and minimal cycle length variability. Initially, a 2:1 AV relationship was observed, which subsequently progressed through 3:2, 4:3, and 5:4 AV relationships, ultimately leading to a 1:1 AV relationship **(Figure 3)**. Ventricular entrainment maneuvers yielded a long post-pacing interval and a long stimulus-to-atrial–VA interval, suggesting specific re-entrant circuit properties **(Figure 2B)**. A His-synchronous ventricular extrastimulus did not perturb the tachycardia, further supporting the involvement of the AV node in the re-entrant circuit. The presence of a dual AV nodal physiology, along with the entrainment findings and a short VA tachycardia, collectively indicated a diagnosis of typical AVNRT. Notably, the observed Wenckebach periodicity was driven by a gradual prolongation of the A–H interval, rather than the H–V interval, preceding the absence of ventricular activity **(Figure 4)**. The H–V and atrial–atrial (A–A) intervals remained fixed and similar. A His-bundle potential (HBP) was consistently missed when a ventricular beat was also missed.

Consent was obtained from the patient in line with the Committee on Publication Ethics (COPE) guidance.

## Discussion

A meticulous electrogram analysis was conducted to elucidate the sequence of events leading to a dropped beat.

### Observations preceding the dropped beat

In the cardiac cycle immediately preceding the dropped beat, a crucial finding was the temporal relationship between the HBP and the atrial signal across all coronary sinus electrograms **(Figures 3 and 4)**. The HBP was consistently observed after the atrial deflection. This configuration signifies a maximal A–H interval prolongation, which was sufficient to place the HBP subsequent to the retrograde atrial activation. This sequence suggests that the maximum possible A–H delay had been achieved before the conduction failure. The observed progressive A–H interval prolongation, which defines the Wenckebach periodicity, must be attributed to a conduction decrement within the LCP rather than the antegrade slow pathway (SP). Had the Wenckebach phenomenon occurred in the antegrade SP, the resultant block would have interrupted the forward limb of the re-entrant circuit, thereby terminating the tachycardia. The sustained nature of the rhythm, despite the progressively delaying conduction, confirms that the site of block lies distally in the LCP (infranodal, suprahisian Wenckebach), allowing the re-entrant circuit to persist despite having an LCP block.

### Sequence following the dropped beat

The dropped QRS complex was attributed to a block in the LCP as discussed earlier **(Figures 1 and 2)**. Immediately following this LCP block, the HBP timing reverted, appearing before the atrial signal as seen in conventional AVNRT.

### Conduction dynamics

The progressive delay observed in the LCP conduction resulted in the HBP exhibiting temporal overlap with the atrial activity within the His catheter recording. This overlap significantly impeded the clear discrimination and measurement of the HBP from the atrial signal in the His catheter.

Successful SP ablation led to the complete abolition of inducible tachycardia, even under aggressive pacing protocols and with isoprenaline challenge. Post-procedure electrophysiological study revealed normal A–H and H–V intervals, with an AV Wenckebach cycle length of 350 ms.

The occurrence of a 2:1 AV relationship during AVNRT is a well-established phenomenon, observed in up to 10% of inducible AVNRT cases during electrophysiological studies.^1,2^ While ablation of the AV nodal junctional tissue (SP region) effectively abolishes re-entry, the precise anatomical and electrophysiological circuit of AVNRT remains a subject of ongoing investigation. The documented presence of atrial or ventricular dissociation during ongoing AVNRT substantiates that ventricular or atrial tissue is not an absolute requirement for the sustenance of this re-entrant tachycardia. This supports the classification of AVNRT as an intranodal re-entry, with the proposed involvement of an upper common pathway (UCP) or an LCP. However, recent evidence has raised doubts about the universal presence of the UCP.^4^

The continuation of AVNRT in the absence of His and ventricular activity supports the notion of an LCP. The AV nodal tissue between the typical AVNRT circuit and the His bundle constitutes an LCP. This is further substantiated by the finding that the His-to-atrial interval during para-Hisian entrainment is longer than that during AVNRT itself.^5^ It has been suggested that an LCP may be present in up to 78% of AVNRT patients. The presence of an HBP preceding a blocked ventricular beat suggests either an intra- or infrahisian block. Our patient uniquely presented with a gradual prolongation of the A–H interval (with fixed A–A and H–V intervals) during AVNRT, followed by a dropped QRS complex and a return to a shortened A–H interval to repeat the cycle. Although low atrial tachycardia remains a rare possibility that could present with a similar Wenckebach phenomenon, the totality of corroborative electrophysiological evidence—including dual AV nodal physiology, tachycardia induction property, characteristic entrainment findings, and the definitive abolition of the tachycardia upon ablation of the SP region—strongly supports AVNRT as the most plausible and definitive diagnosis.

## Conclusion

In conclusion, we present a rare case of AVNRT characterized by spontaneous LCP Wenckebach periodicity. This phenomenon was defined by fixed and similar A–A and H–V intervals with a progressively lengthened A–H interval preceding a dropped ventricular beat. The crossover of the HBP over atrial electrogram suggests an LCP block and strongly supports a supra-Hisian infranodal block as the mechanism underlying the Wenckebach periodicity.

## Data Availability

All data are incorporated into the article and its online supplementary material.
